# Characterization of a Decellularized Sheep Pulmonary Heart Valves and Analysis of Their Capability as a Xenograft Initial Matrix Material in Heart Valve Tissue Engineering

**DOI:** 10.3390/bioengineering10080949

**Published:** 2023-08-09

**Authors:** Müslüm Süleyman İnal, Cihan Darcan, Ali Akpek

**Affiliations:** 1 Department of Molecular Biology and Genetics, Institute of Science, Bilecik Seyh Edebali University, Bilecik 11230, Turkey; 3917026@ogrenci.bilecik.edu.tr; 2 Department of Molecular Biology and Genetics, Faculty of Science, Bilecik Seyh Edebali University, Bilecik 11230, Turkey; cihan.darcan@bilecik.edu.tr; 3 Department of Biomedical Engineering, Faculty of Electrical-Electronics, Yildiz Technical University, Istanbul 34220, Turkey

**Keywords:** decellularization, heart valve, tissue engineering, xenograft, biomaterial

## Abstract

In order to overcome the disadvantages of existing treatments in heart valve tissue engineering, decellularization studies are carried out. The main purpose of decellularization is to eliminate the immunogenicity of biologically derived grafts and to obtain a scaffold that allows recellularization while preserving the natural tissue architecture. SD and SDS are detergent derivatives frequently used in decellularization studies. The aim of our study is to decellularize the pulmonary heart valves of young Merino sheep by using low-density SDS and SD detergents together, and then to perform their detailed characterization to determine whether they are suitable for clinical studies. Pulmonary heart valves of 4–6-month-old sheep were decellularized in detergent solution for 24 h. The amount of residual DNA was measured to determine the efficiency of decellularization. Then, the effect of decellularization on the ECM by histological staining was examined. In addition, the samples were visualized by SEM to determine the surface morphologies of the scaffolds. A uniaxial tensile test was performed to examine the effect of decellularization on biomechanical properties. In vitro stability of scaffolds decellularized by collagenase treatment was determined. In addition, the cytotoxic effect of scaffolds on 3T3 cells was examined by MTT assay. The results showed DNA removal of 94% and 98% from the decellularized leaflet and pulmonary wall portions after decellularization relative to the control group. No cell nuclei were found in histological staining and it was observed that the three-layer leaflet structure was preserved. As a result of the tensile test, it was determined that there was no statistically significant difference between the control and decellularized groups in the UTS and elasticity modulus, and the biomechanical properties did not change. It was also observed that decellularized sheep pulmonary heart valves had no cytotoxic effect. In conclusion, we suggest that the pulmonary valves of decellularized young Merino sheep can be used as an initial matrix in heart valve tissue engineering studies.

## 1. Introduction

Surgical treatment of heart valve diseases is performed in the form of repair or replacement of the valve, depending on the severity of the disease. The method used varies according to valve type, pathology, and disease severity [1]. Operations in which the valve is repaired are called valvuloplasty and are divided into three groups: commissurotomy, annuloplasty, and cordoplasty [1,2,3]. However, in cases where the repair does not work or there is stenosis, insufficiency, or both, replacement therapy is the only solution. Although these diseases are of genetic or environmental origin, modified valves, they are mechanical heart valves that do not have a growth feature and require lifelong anticoagulant medication, or short-lived bioprosthetic valves that can create an immune response in patients [4,5]. Heart valve tissue engineering is promising to overcome these disadvantages of current treatment methods [6].

In tissue engineering, attempts have been made to produce tissue engineering heart valves using natural polymers such as collagen, hyaluronic acid, alginate, or gelatin, and synthetic polymers such as polycaprolactone, polyglycolic acid, and poly L-lactic acid, by printing methods such as 3D bioprinting, stereolithography, and electrospinning [7,8,9,10]. However, it has been reported that the artificial heart valves obtained due to the defects such as secondary structure change, great variability from batch to batch, and inadequate mechanical properties of the polymers used could not show sufficient efficiency in terms of both functional and bioresistance [11,12]. Therefore, decellularization studies of grafts of biological origin are carried out in order to obtain a scaffold that can be used as a starting matrix in the field of tissue engineering. In addition to having a natural ECM architecture, bioprosthetic valves are immunogenic, so cells are removed with decellularization applications, and acellular, porous scaffold remains [13]. In heart valve tissue engineering, two different sources can be used: xenograft (graft obtained from different species) or allograft (graft obtained from the same species). The use of allografts as a source when designing human heart valves is limited to cadavers or organ donors. Therefore, xenografts without resource problems seem more attractive. However, the presence of xenoantigens found in the ECM after decellularization in xenografts and creating an immune response in humans may adversely affect graft success [14]. Therefore, the decellularization method, density, and subsequent characterization processes are of vital importance.

Decellularization methods are divided into chemical, physical, and biological (enzymatic) classes. Physical methods used in decellularization processes include mechanical forces, freezing and thawing, sonication, and radiation. Only cells close to the surface of the tissues can be effectively removed by mechanical forces. During freezing and thawing, intracellular ice crystals disrupt cell membranes, although chemical or enzymatic approaches must be used to remove intracellular materials [11,15]. Trypsin and endonuclease enzymes are generally used in enzymatic methods. While trypsin facilitates cell removal, it also affects the fibrous structural proteins of the valve ECM, causing damage to the histoarchitecture [16]. Chemical decellularization processes are mostly available in the literature, since physical and enzymatic approaches alone are not sufficiently effective in cell removal [17]. Chemical methods have been frequently studied to develop nonimmunogenic and long-lasting grafts in heart valve tissue engineering [18,19]. Examples of chemical methods are acid/base solutions, hypotonic/hypertonic solutions, and ionic/nonionic detergents. Especially, ionic/nonionic detergents are mostly used in decellularization studies due to effective cell removal [16]. Triton X-100, sodium deoxycholate (SD), sodium dodecyl sulfate (SDS), and ethylenediaminetetraacetic acid (EDTA) are the most widely used detergents for decellularization. Except for low doses, SDS has been reported to cause degeneration of histoarchitecture and a cytotoxic environment for cells [20]. There are studies reporting that SD completely removes cells while preserving the histoarchitecture [21,22]. Triton X-100, which is one of the nonionic detergents, is a detergent that is frequently used in decellularization studies and is effective in cell extraction, DNA removal, and also in the protection of extracellular matrix components [23,24]. EDTA promotes cell dissociation without causing ECM damage, but is not effective in cell removal alone [25]. Therefore, it is applied in combination with other detergents or enzymatic treatments [25,26]. In our study, inspired by previous studies, it was decided to use both SD, which does not harm the extracellular matrix (ECM) histoarchitecture and has proven efficacy in decellularization, and SDS, which is very effective in cell removal and does not have a low-concentration cytotoxic effect [27,28,29,30,31].

Pigs are generally used as donors in decellularization studies of heart valve tissue engineering. Among the reasons for this, it can be said that the biomechanical properties are good and the anatomy is similar to human valves [32]. However, considering the risk of porcine endogenous retrovirus (PERV) infection from porcine heart valves [33], the use of glutaraldehyde, which has a cytotoxic effect as a stabilizing agent [34], and the immunogenic effect in almost all studies according to clinical findings [35,36,37], a different source of xenografts should be found as an alternative to pigs. Decellularized sheep heart valves have been tested in in vivo implant models such as sheep, pig, and rabbit [38,39,40,41]. It has been observed in animal trials that decellularized sheep heart valves do not induce an inflammatory response, and recellularization occurs [42,43,44]. However, no clinical trial of decellularized sheep heart valves has been found. Virtually all characterizations of decellularized porcine heart valves have been performed; in vivo trials, clinical trials, and even commercial products have been created [31,35,45,46].

In the few studies in the literature on decellularized sheep heart valves, in vitro recellularization trials of sheep heart valves without detailed characterization after decellularization have been investigated for calcification tendencies and immunogenicity in various implant models [30,47,48,49]. Tudorache et al. (2013) decellularized sheep heart valves and then implanted them in sheep to investigate their function and morphological changes [47]. In this study, a detergent-based method as well as cryopreservation procedures were applied for the decellularization process and characterizations were performed after transplantation into sheep to evaluate their efficacy. It was emphasized that the efficacy of the decellularized group after transplantation was good in the early term, but it was reported that cryopreserved samples caused calcification and immune response. However, the main purpose of heart valve tissue engineering is to design human-specific valves. The use of sheep as an implant model was originally made to determine allograft effectiveness. Therefore, detailed characterization procedures are required first for human implantation of decellularized sheep valves. Theodoridis et al. (2015) investigated the matrix-guided tissue regeneration potential of decellularized sheep heart valves in aged sheep as a transplantation model [30]. Since allografts were used in this study, detailed characterization processes for humans after decellularization were not performed. Converse et al. (2017), in a published study, after processing sheep aortic heart valve with detergent-based decellularization, tried recellularization with human mesenchymal stem cells under bioreactor conditions without characterization processes and reported that they could not achieve full cellularity [49]. As can be seen, inadequate or no characterization of decellularized sheep heart valves may cause such problems. Therefore, detailed characterization tests are needed to evaluate sheep heart valves as a starting matrix in tissue engineering studies.

Our aim in this study is to characterize sheep heart valves, which are not yet ready for clinical trials, after decellularization with a detergent-based chemical method. In the Ross procedure, pulmonary autograft valves are implanted in place of damaged aortic valves in pediatric and young patients [50]. It is also possible to use them orthotopically in damaged pulmonary cases. Due to their versatility, it was decided to investigate pulmonary valves in our study. Initially, pulmonary heart valves of 4–6 month old merino sheep were decellularized using ionic detergents such as SD and low concentration SDS. The efficiency of decellularization was determined by determination of residual DNA amount, histological staining, scanning electron microscopy (SEM) imaging, determination of swelling ratio, and uniaxial tensile test. Thanks to our characterizations, it will be possible to determine the suitability of sheep pulmonary heart valves decellularized by a detergent-based method as xenograft for clinical studies.

## 2. Experimental Design

### 2.1. Decellularization

Hearts collected from a local slaughterhouse, approximately 20 min after the slaughter of 4–6 month old merino sheep, were brought to the laboratory in cold saline (0.9%). In a sterile environment, pulmonary valves were isolated from hearts and cleared of external connective tissue and adipose tissue. They were then shaken in 1× PBS (Sigma, St. Louis, MO, USA, 524650) solution containing 0.8% SD (Sigma, 30970) and 0.2% SDS (Sigma, 71725) at 37 °C for 24 h [51]. They were washed 6 times for 12 h each with PBS containing streptomycin and penicillin (100 IU/mL) (Gibco, Waltham, MA, USA, 15140-122). Decellularized scaffolds were incubated with solutions of DNase I (200 µg/mL, Biomatik, ON, Canada, A2442) and RNase A (50 µg/mL, Biomatik, ON, Canada, A3806) prepared in 10 mM MgCl_2_ and 50 mM Trisma (pH: 7.5) buffer for 24 h at 37 °C. After this time, they were washed with several rounds of ultrapure water. After washing, decontamination (with 70% ethanol) was applied for 5 h. Decellularized scaffolds and nondecellularized native valves as a control were kept in PBS solution containing streptomycin and penicillin (100 IU/mL) at 4 °C until characterization was completed.

### 2.2. Residual DNA Measurement

Manual DNA isolation [52] was performed to determine the amount of residual DNA after decellularization. Sections of 1 cm × 1 cm were taken from the pulmonary wall and leaflet parts of the control group and decellularized scaffolds (*n* = 3) under sterile conditions. The samples were kept in a solution containing 10 mM Trisma, 50 mM KCl, 1.5 mM MgCl_2_, 0.5% Tween 20, and 20 mg/mL Proteinase K (NZYTech, Lisbon, Portugal, MB01902) at 55 °C for 48 h. Then, the solution was centrifuged at 3000× *g* at +4 °C for 15 min and the supernatant was collected. Phenol: chloroform: isoamyl alcohol (25:24:1 by volume, respectively) was added to the supernatant and centrifuged at +4 °C at 3000× *g* speed for 15 min. A total of 200 μL of 3 M sodium acetate (pH: 5.5) and 500 μL of 95% ethanol were added to the collected supernatant and incubated at −80 °C for 20 min. Afterwards, it was incubated at 37 °C for 30 min and centrifuged at 10,000 rpm at 25 °C for 10 min and the supernatant was discarded. After adding 100 μL of distilled water on the remaining pellet and dissolving the DNA, the amount of double-stranded DNA (260/280 nm) was measured with nanodrop (SHIMADZU, Columbia, SC, USA, BioSpec-nano) and compared with the nondecellularized control group. Data were found in ng/μL and DNA removal was calculated as %.

### 2.3. Histological Characterization

Histological staining was performed to observe the effects of decellularization on the pulmonary wall and leaflet portions and to confirm acellularity. As a preliminary preparation, decellularized leaflet and wall samples and control group samples were fixed with 10% neutral buffered formaldehyde solution. Then, dehydration was achieved by passing through increasing ethanol series (60–70–80–90–96–100). After the samples were embedded in paraffin, they were cut into 5 μm sections with a microtome (Leica, Wetzlar, Germany, RM2245). They were then treated with xylene after incubation at 55 °C to remove paraffin from the samples. After the deparaffinization process, rehydration was achieved by passing the samples through decreasing series of ethanol. Subsequently, the samples were stained with H&E (Vector, Olean, NY, USA, H3502) and Movat pentachrome (Abcam, Cambridge, UK, ab245884). H&E stains the cytoplasm of cells and ECM proteins pink, while the nucleus stains blue–violet. Movat pentachrome also stains collagen yellow, elastin black, glycosaminoglycans blue, and nuclei black. Following the staining process of the preparations, they were observed with a light microscope (Olympus, Shinjuku City, Tokyo, Japan, BX53) and their images were recorded.

### 2.4. SEM Imaging

Scanning electron microscopy (SEM) (Zeiss EVO, Jena, Germany, LS10) was used to observe the surface morphology of the pulmonary leaflet and wall samples of the control and decellularized groups. The samples were fixed for at least 24 h with a solution containing 2.5% by volume glutaraldehyde prepared in PBS. Then, the scaffolds were washed with ultrapure water and passed through increasing series of ethanol (60–70–80–90–96–100%) for 5 min each and left to dry at room temperature. The dried scaffolds were coated with gold (80%)–palladium (20%) by the spray coater for 90 s. Then, imaging was started with SEM at 15.00 kV 1000× magnification.

### 2.5. Tensile Test

A uniaxial tensile test was performed to determine the effect of decellularization on sheep heart valve biomechanical properties. After the pulmonary wall and leaflet parts of the control group and decellularized scaffolds (*n* = 3) were moistened with PBS, they were cut into 1–2 cm strips and placed in a general tensile testing device (Instron-3367). They were stressed at a rate of 5 mm/min with an initial load of 0.1 MPa at room temperature [53]. The initial thickness, length, and width of the samples were also noted, and the final tensile strength (UTS) and modulus of elasticity (Young’s modulus) values were recorded with the data obtained after the test.

### 2.6. Collagenase Degradation Experiment

Native and decellularized pulmonary heart valves (*n* = 3) were cut to 1 cm × 1 cm dimensions and the initial weight (*W*_0_) of each sample was determined after lyophilization (Labart, LFD-10N). Collagenase I (Gibco-17100017) solution was prepared in PBS buffer at a concentration of 1 mg/mL (125 U/mL). A total of 1 mL of collagenase solution was added to each centrifuge tube with the samples and incubated at 37 °C at 60 rpm for 24 h under shaking condition. The tubes were then centrifuged at 10,000 rpm for 15 min and the supernatant was slowly poured out. The samples were lyophilized after washing with PBS. After completely removing the water, the samples were weighed again (*W*_1_) and the degradation rate (%) was calculated with the following formula [54].

$$\%\;Degredation\;Rate=\frac{W_{0}-W_{1}}{W_{0}}\times 100$$

### 2.7. Swelling Ratio

Pieces (*n* = 3) of 1 cm × 1 cm were cut from the pulmonary wall and leaflet parts of the control group and decellularized samples, and their initial dry weights were weighed. The cut samples were dipped into tubes containing PBS. Samples kept in the tube at 37 °C for 4 h were collected and excess PBS was carefully wiped away. The final weights were then weighed and the % swelling ratio was calculated according to the formula [53]

$$\%\;swelling\;ratio=\left(\frac{Wet\;weight-Dry\;weight}{Dry\;weight}\right)\times 100$$

### 2.8. In Vitro Cytotoxicity Evaluation

Decellularized leaflet and pulmonary wall samples were cut into 1 cm × 1 cm dimensions (*n* = 3) and sterilized with 75% ethanol for 24 h. They were then washed 10 times with sterile 1× PBS (CAPRICORN, PBS-1A). Samples were transferred to Dulbecco’s Modified Eagle’s Medium (DMEM) (CAPRICORN, DMEM-HA) for 24 h in an incubator (Nüve EC-150) at 37 °C at 5% CO_2_. The extract was then collected for cell culture. NIH 3T3 (CRL-1658-ATCC, Manassas, VA, USA) cells were seeded in a 96-well plate at a density of 7 × 10^3^ per well and DMEM containing 10% FBS (CAPRICORN, Ebsdorfergrund, Germany, FBS-11B) and 1% penicillin/streptomycin was used as culture medium. After the cells adhered to the plate, the cell culture medium was removed and replaced with 200 µL of the extract. Cell viability was measured using an MTT (Solarbio, M8180, Beijing, China) assay kit at 3 different time periods: 24 h, 48 h, and 72 h, according to the manufacturer’s instructions. Cells to which no extract was added and that were grown in DMEM were determined as the control group [55].

### 2.9. Statistical Analysis

Data are given as mean ± standard deviation. IBM SPSS Statistics (Version 23) software was used for statistical analysis. One-way ANOVA test was used to compare the means of groups. A value of *p* < 0.05 was considered statistically significant.

## 3. Results and Discussion

### 3.1. Swelling Ratio

**Figure 1:**
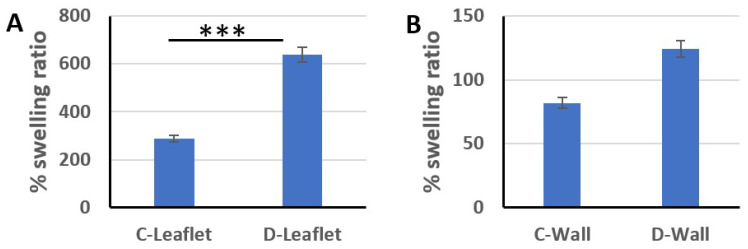
% swelling ratios of control and decellularized leaflet (**A**) and pulmonary wall samples (**B**). *** *p* < 0.001 is statistically significant (C: control, D: decellularized).

The swelling rate of the scaffolds was analyzed to determine the effectiveness of decellularization. With decellularization applications, the cells separated from the ECM are replaced by spaces and the porosity increases. Therefore, the swelling rate of the scaffolds increases. As a result of our measurements, the % swelling rate of leaflet samples in the control group was 288.4 ± 85.6, while this rate increased to 638.5 ± 60 after decellularization. The swelling rate of the pulmonary wall specimens of the control group was 81.9 ± 16.3 and increased to 124.3 ± 9.3 after decellularization (Figure 1). Compared to the control (native) group, the % swelling rates of the decellularized leaflet and wall samples were increased by 121% and 52%, respectively.

### 3.2. Residual DNA Content

**Figure 2:**
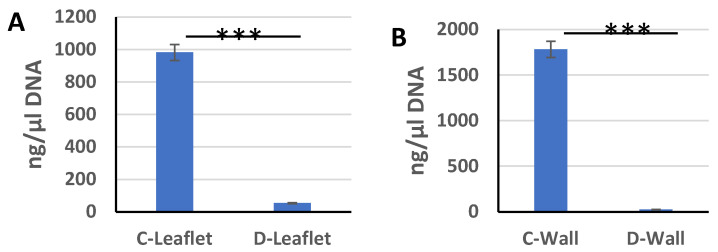
Nanodrop measurement results of DNA obtained from control group and decellularized leaflet (**A**), pulmonary wall (**B**) samples (ng/µL). *** *p* < 0.001 is statistically significant.

In order to determine the residual DNA amount, DNA isolation was performed and the measurement results in ng/µL were obtained with nanodrop. According to the nanodrop measurement results, the DNA amount of the leaflet samples of the control group was 982 ± 131.3 ng/µL, and 1783.3 ± 110.4 ng/µL in the pulmonary wall samples. After decellularization, the amount of double-stranded DNA in leaflet samples decreased to 54.22 ± 9 ng/µL and the amount of DNA in pulmonary wall samples decreased to 26.04 ± 13 ng/µL (Figure 2). DNA removal in decellularized samples compared to control groups was 94% and 98% in leaflet and wall parts, respectively. In most of the studies in the literature, approximately 95% of DNA is cleared by decellularization [44,56]. In our study, DNA removal was similar to the literature data and detergent-based decellularization process with at least 94% DNA removal provided effective decellularization in sheep heart valves. In order to say that the decellularization process is effective, it has been reported that in addition to the high level of DNA removal, no cell nuclei and stained cells should be detected in histological stainings [57].

### 3.3. SEM Imaging

**Figure 3:**
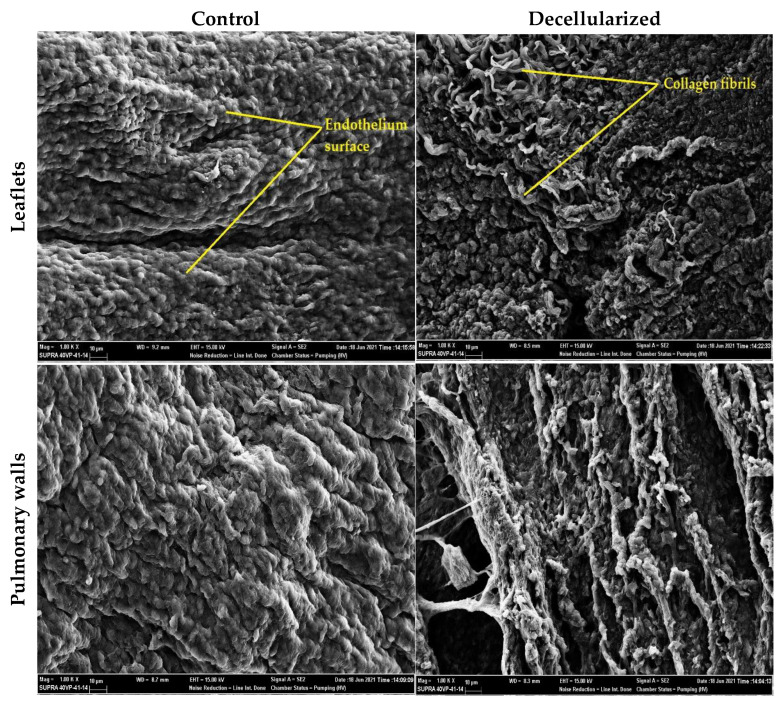
SEM image (1000×) of control and decellularized leaflet/wall samples. An almost smooth endothelial surface was observed in the control groups, whereas decellularized samples showed a porous surface where the endothelium was removed and fibrils were exposed.

In SEM images, a patterned endothelial layer is seen on the surface of leaflet and pulmonary wall samples of the control group (Figure 3). In the images of decellularized leaflet and wall samples, it is observed that the endothelial layer has disappeared and the complex fibrous networks underneath are exposed [58,59]. There is no significant change in the orientation of the exposed collagen fibrils. In addition, based on these images, no cells were found near the surface after decellularization. In addition, the space remaining after cell removal is also clearly visible. Therefore, according to the SEM images, the cell extraction process was performed successfully and there was no major change in the ECM.

### 3.4. Histological Assessment

**Figure 4:**
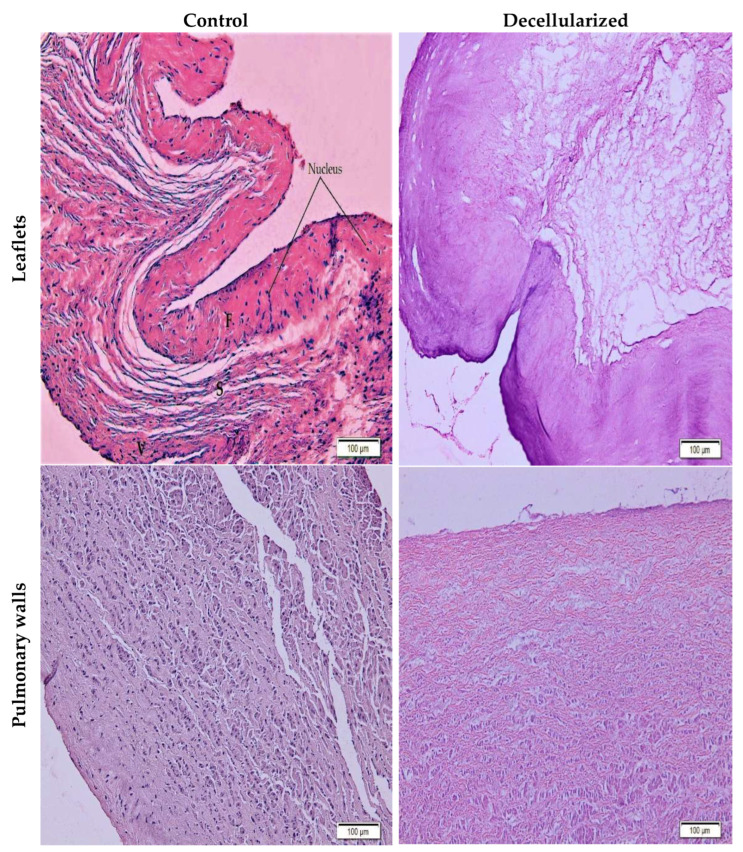
H&E stained control and decellularized leaflet/wall images (20× magnification); F: fibrosa, S: spongiosa, V: ventricularis layers. While black-stained nuclei were observed in the control groups, no nuclei were found in the samples after decellularization.

The leaflets are composed of three layers: fibrosa, spongiosa, and ventricularis, respectively. Predominantly, collagens are located in the fibrosa layer, glycosaminoglycans in the spongiosa layer, and elastins in the ventricularis part. Depending on the method used in the decellularization process, the basic ECM components in this three-layer structure are slightly affected. To determine this damage, H&E and Movat pentachrome staining were performed. According to the H&E staining images, it was shown that there was no difference in the amount and orientation of collagen in the fibrosa layer of the leaflets (Figure 4).

**Figure 5:**
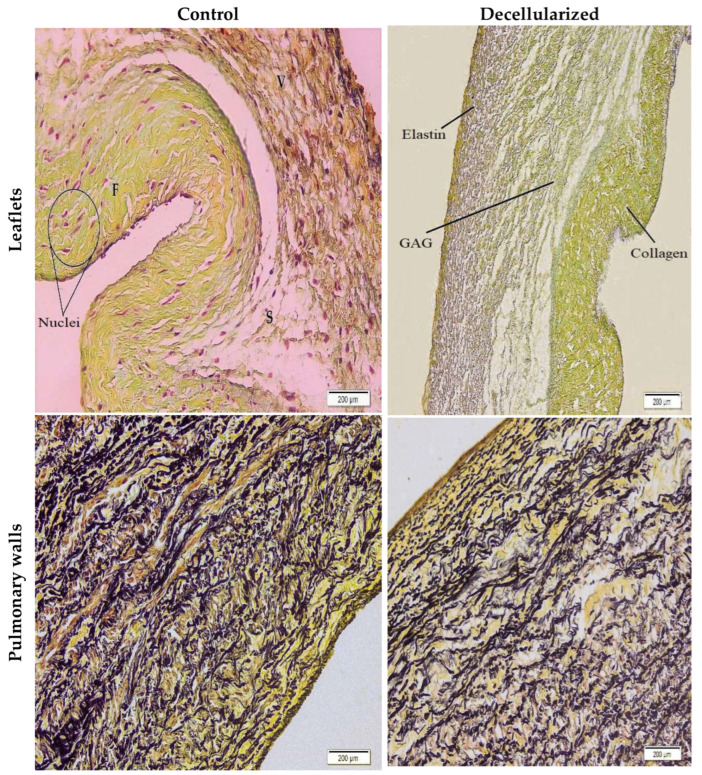
Movat’s pentachrome stained control and decellularized leaflet/wall images (10× magnification). While black-stained elastins and nuclei, yellow-stained collagens, and blue-stained GAGs were visible in the control groups, no nuclei remained after decellularization and other ECM components were preserved.

No significant difference was observed in terms of collagen arrangement and amount in the three layers of leaflets stained with Movat pentachrome after decellularization compared to the control group (Figure 5). It is known that the decellularization process leads to collagen reduction in these layers [60]. However, when compared with the control group, both leaflets and walls appear to be similar in terms of collagen organization and amount [61]. In addition, the amount of elastin was slightly affected by the decellularization process in the pulmonary wall sections, but no critical change occurred. In a study investigating the change of glycosaminoglycans (GAG), which is located in the spongiosa layer of heart valves obtained from humans and sheep, which is effective in the viscoelastic behavior of tissues after decellularization, a report was presented that showed greater loss of GAG in sheep leaflets, and this was due to the greater initial cellularity in sheep leaflets [62]. In the same study, according to the quantitative measurement results of sulfated GAGs, a decrease of approximately 89.6% was observed in sheep heart valves after decellularization, while this rate was 57.4% in human valve leaflets. In addition, no significant change was observed in the blue-stained GAGs after decellularization in our study.

No stained nuclei or cellular residues were found in the images obtained as a result of H&E and Movat pentachrome staining. Although the three-layer leaflet structure was preserved, there was no significant change in basic ECM components such as collagen, elastin, and GAG (Figure 4 and Figure 5). Cell extraction was successfully performed in the pulmonary leaflet and wall portions decellularized with SD and low-intensity SDS, as SEM images also confirmed the absence of endothelium on the surface.

### 3.5. Tensile Test

**Figure 6:**
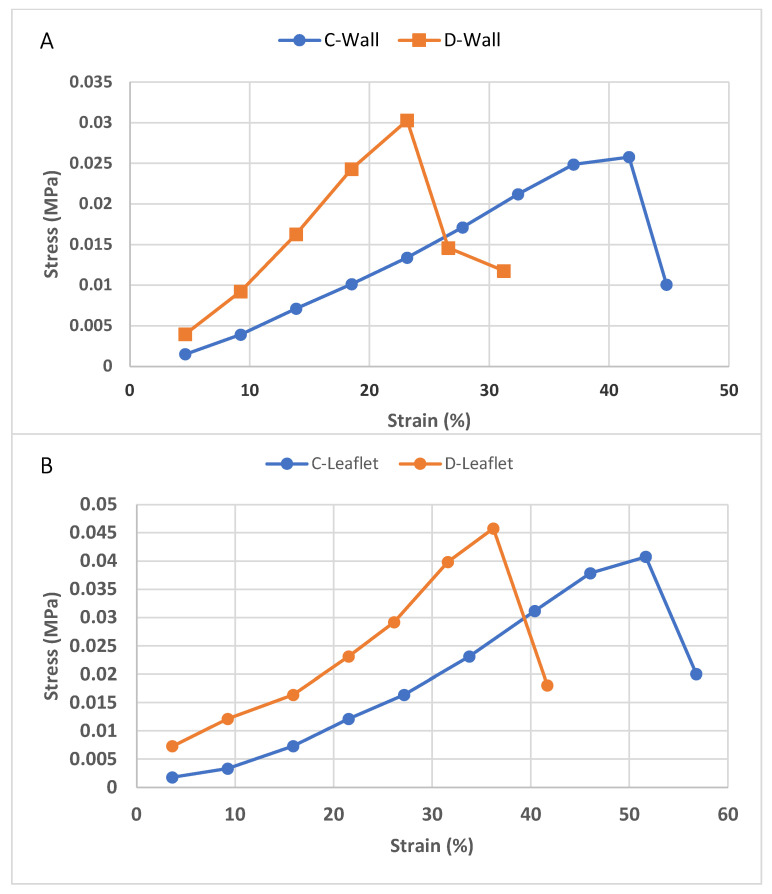
Tensile stress and strain curve of control group and decellularized pulmonary wall (**A**) and leaflet (**B**) samples.

To examine the effect of decellularization processes on the biomechanical properties of tissues, the ultimate tensile strength (UTS) and modulus of elasticity (Young’s modulus) of pulmonary wall and leaflet sections taken in the longitudinal direction were analyzed in a conventional tensile testing device. Looking at the stress and strain curve, it is observed that stiffness increases and elongation decreases with decellularization in both leaflet and pulmonary wall samples (Figure 6). As with the histological images, it is likely that the extensibility of the samples is also slightly reduced due to the reduced crimping of the collagen fibers after decellularization [63]. However, according to the data obtained, it was observed that there was no statistically significant change in the UTS and modulus of elasticity values (Table 1). It has been reported that after detergent-based decellularization, there is no change in stretching properties, especially UTS, in sheep heart valves compared to the control group [59]. As observed in histological staining, minimal changes in collagen, elastin, and GAG components and preservation of the 3D architecture ensured the preservation of mechanical properties. Similar findings were obtained in porcine heart valves, whose biomechanical properties are known to be preserved after decellularization, and no significant differences in tensile properties were observed when compared with control groups [58]. The tensile test alone is not sufficient to examine the effect of decellularization on the biomechanical properties of heart valves. Therefore, there is a need to perform functional tests such as fatigue testing under physiological conditions. In our future studies, these tests will be performed to determine the durability and compatibility of heart valves after decellularization.

### 3.6. Collagenase Degradation

**Figure 7:**
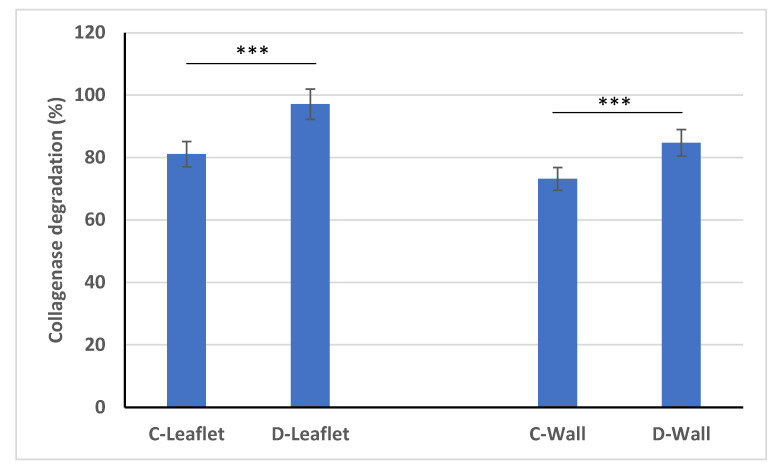
Comparison of the enzymatic degradation profiles of decellularized leaflet and pulmonary wall samples with their native counterparts. *** *p* < 0.001 is statistically significant.

In order to examine the stability of decellularized and native leaflet and wall samples, they were exposed to degradation by collagenase enzyme for 24 h (Figure 7). In order to examine the stability of decellularized and native leaflet and wall samples, they were exposed to degradation by collagenase enzyme for 24 h. At the end of this period, it was observed that the leaflet samples were almost completely degraded. The wall samples, on the other hand, partially preserved their integrity. While the degradation rate of natural leaflet samples was 81.14 ± 2.29%, the degradation rate of leaflets after decellularization was determined as 97.16 ± 2.12%. Similarly, while the degradation rate of the native pulmonary wall sample was 73.2% ± 1.45%, this rate increased to 84.76 ± 2.02% after decellularization. With the decellularization process, the in vitro stability of both leaflet and wall samples decreased compared to their natural counterparts. Similarly, in a study conducted in 2019, porcine pulmonary valves were decellularized using a detergent-based method, and degradation rates after collagenase treatment were determined by Fourier transform infrared spectroscopy [64]. According to the results of this study, it was understood from the infrared absorption spectra that the decellularized leaflets were completely degraded, and that the pulmonary wall samples, especially the outer layer, were more degraded than the inner layers. These results demonstrate the importance of in vitro recellularization of decellularized heart valves. As a result, collagen, which is the main protein of ECM, is exposed by the decellularization process and cannot resist enzymatic degradation. In addition, there are studies in which the degradation rate is significantly reduced by making various modifications of the ECM after decellularization [29,54]. Biomechanical properties and in vitro stability of decellularized sheep heart valves can be improved by these methods.

### 3.7. In Vitro Cytotoxicity

**Figure 8:**
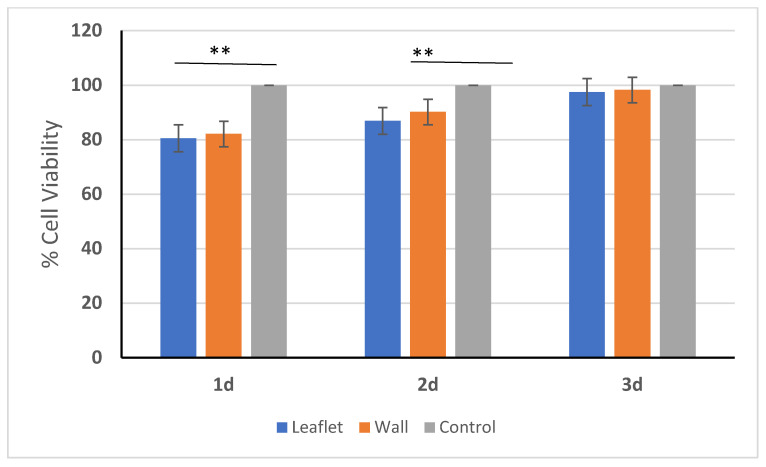
Cell viability in decellularized leaflet and pulmonary wall and comparison with control group. Only fresh medium was used in the control group. ** *p* < 0.01 is statistically significant.

An in vitro cytotoxicity assay was performed to evaluate the biocompatibility of decellularized pulmonary valves against possible detergent residue. According to MTT findings, no cytotoxic effect of the extract obtained from decellularized leaflet and pulmonary wall samples was observed on 3T3 cells, and cell viability increased day by day (Figure 8). Although SDS is a widely used detergent in the decellularization process, it has been reported to cause ECM damage depending on the concentration, while at the same time it has a cytotoxic effect on cells as a residual detergent [65]. However, there is also evidence that low-concentration SDS does not cause ECM damage and provides complete decellularization [66]. In a study by Luo et al. conducted in 2019, they decellularized porcine aortic heart valves between 0.5% and 2% with different concentrations of SDS and showed that no sample had a cytotoxic effect on 3T3 cells in their in vitro cytotoxic analysis [55]. Similarly, in our study, low-dose SDS-containing detergent-based decellularization and continuous agitation conditions ensured effective removal of detergents from the ECM, and cytotoxicity was not observed.

Similar studies were performed on porcine heart valves, and their general findings are given in Table 2 for comparison purposes. In general, the decellularization process does not cause a significant change in the biomechanical properties of either pig or sheep valves. In addition, especially combining detergent- and enzymatic-based treatments is effective in DNA removal. Also, if decellularized valves are not fixed [67], as in our study, their resistance to enzymatic degradation is reduced and they are almost completely hydrolyzed regardless of the donor type.

## 4. Conclusions

In this study, sheep pulmonary heart valve was decellularized using SD and low-density SDS to obtain a starting matrix suitable for applications in heart valve tissue engineering. The scaffolds obtained were subjected to various characterizations in order to determine the efficiency of this process following decellularization and to determine the changes occurring in the samples. Histological images, % swelling ratio, which indirectly indicate porosity, and residual DNA analysis results showed us that we obtained a completely acellular scaffold. In addition, SEM and histological images showed that the natural architecture and three-layer leaflet structure were significantly preserved. In the tensile test for biomechanical characterization, it was observed that there was no significant change after decellularization and the detergent-based decellularization method provided efficient cell removal. In addition, it was determined that the decellularized scaffolds had no cytotoxic effect in the in vitro cytotoxicity test. It was also observed that the in vitro stability of decellularized scaffolds decreased after treatment with collagenase. Although porcine heart valves have been used in most of the studies in the literature, such detailed characterization tests of decellularized sheep heart valves have not been performed together. Our study, for the first time, shows, through many different characterizations, that merino sheep heart valves are suitable for recellularization, have a porous structure, and do not have critical changes in mechanical properties. In our future studies, we plan to recellularize the decellularized sheep heart valves with human cells in vitro and investigate their regeneration potential in this scaffold. As a result, this study finally proves that decellularized sheep heart valves are a promising candidate in heart valve tissue engineering as a candidate for xenograft, and baseline values that can be considered as reference values as a starting matrix were revealed.

## Figures and Tables

**Table 1:** Tensile test results of control group and decellularized samples. Since *p* > 0.05, there is no statistically significant difference between groups.

	UTS (MPa)	Modulus of Elasticity (MPa)
C-Leaflet	0.041 ± 0.02	0.0042 ± 0.0017
D-Leaflet	0.049 ± 0.01	0.0023 ± 0.001
C-Wall	0.033 ± 0.02	0.0037 ± 0.002
D-Wall	0.047 ± 0.015	0.011 ± 0.003

**Table 2:** General findings of studies with porcine heart valves.

Heart Valve Type	Decell. Agents	General Finding	Reference
Porcine aortic valve	Trypsin-EDTA + Triton X100 + SD	DNA removal after decellularization was 88.3%, exposed fibers seen in SEM images, the modulus of elasticity in the circumferential sections of the native and decellularized samples were determined as 13.8 kPa and 10.5 kPa, respectively, and no statistically significant difference was found.	[55]
Porcine aortic valve	N-Lauroylsarcosine sodium salt	DNA removal after decellularization was 92%, weight losses of native and decellularized samples after collagenase treatment were 80% and 90%, respectively, the decellularization process did not change the modulus of elasticity, while the values of the native and decellularized samples (circumferential direction) were measured as 12.8 MPa and 13 MPa, respectively.	[68]
Porcine pulmonary valve	Triton X-100 + SDS	DNA was reduced by 66% after decellularization of pulmonary wall samples, the UTS value of the native samples sectioned in the circumferential direction was determined as 0.5 MPa, and a statistically significant increase to 1.2 MPa was observed after decellularization; similarly, a significant increase from 1.2 MPa to 3.5 MPa was observed in the modulus of elasticity.	[69]
Porcine pulmonary valve	Trypsin-EDTA	The UTS values of the circumferential sections of native and decellularized leaflet samples were determined as 8.2 MPa and 7.8 MPa, respectively, while the elastic modulus values were measured as 24 MPa and 25 MPa, respectively, and there was no statistically significant difference between these values.	[67]
Porcine aortic valve	SDS	The UTS values of the circumferential sections of native and decellularized leaflet samples were determined as 4.21 MPa and 1.93 MPa, respectively, while the stiffness values were measured as 7.50 MPa and 3.24 MPa, respectively, and a critical decrease of biomechanical properties was reported after decellularization.	[70]
Porcine aortic valve	SD	DNA removal after decellularization was 93%, the modulus of elasticity of the radial sections of the native and decellularized samples was 3.8 MPa and 3.6 MPa, the UTS values were measured as 1 MPa and 0.7 MPa, respectively, and no statistically significant difference was found.	[71]
Porcine aortic valve	Triton X100 + SD + EDTA	Weight loss in the decellularized group after collagenase II treatment was 100%, while the UTS value of the native samples taken in the circumferential direction was 8.9 MPa; this value almost did not change after decellularization, while the elastic modulus values were measured as 21.8 MPa and 25 MPa, respectively.	[72]
Porcine aortic valve	SD	DNA removal after decellularization was 72%, there was a significant increase in stiffness after decellularization (9.17 to 11.49 N/mm), and there was no significant difference in work to maximum load values.	[73]
Porcine aortic valve	SDS + Triton X100 + SD + EDTA	DNA removal after decellularization was 90%; with Movat’s pentachrome staining, it was observed that collagens and elastins were preserved and the amount of GAG decreased. There was no statistically significant difference in biaxial mechanical properties in both radial and circumferential sections of native and decellularized samples.	[74]
Porcine aortic valve	Triton X-100 + SD + IGEPAL CA-630	Both modulus of elasticity and UTS values in the radial direction of native and decellularized leaflets were found to be approximately 2 MPa, and no significant difference was observed between the two groups. No cell nuclei were found in histological staining.	[45]

## Data Availability

The data presented in this study are available within the article.
